# Nonsense-Mediated mRNA Decay Factor Functions in Human Health and Disease

**DOI:** 10.3390/biomedicines11030722

**Published:** 2023-02-27

**Authors:** Lingling Sun, Justine Mailliot, Christiane Schaffitzel

**Affiliations:** 1 School of Biochemistry, University of Bristol, University Walk, Bristol BS8 1TD, UK; 2 Bristol Engineering Biology Centre BrisEngBio, 24 Tyndall Ave, Bristol BS8 1TQ, UK

**Keywords:** nonsense-mediated mRNA decay, up-frameshift proteins, exon-junction complex, neurodevelopmental disease, cancer, viral NMD evasion

## Abstract

Nonsense-mediated mRNA decay (NMD) is a cellular surveillance mechanism that degrades mRNAs with a premature stop codon, avoiding the synthesis of C-terminally truncated proteins. In addition to faulty mRNAs, NMD recognises ~10% of endogenous transcripts in human cells and downregulates their expression. The up-frameshift proteins are core NMD factors and are conserved from yeast to human in structure and function. In mammals, NMD diversified into different pathways that target different mRNAs employing additional NMD factors. Here, we review our current understanding of molecular mechanisms and cellular roles of NMD pathways and the involvement of more specialised NMD factors. We describe the consequences of mutations in NMD factors leading to neurodevelopmental diseases, and the role of NMD in cancer. We highlight strategies of RNA viruses to evade recognition and decay by the NMD machinery.

## 1. Introduction

Nonsense-mediated messenger RNA decay (NMD) is a conserved eukaryotic pathway that quality controls protein synthesis by recognising and degrading mRNAs bearing premature termination codons (PTCs). One characteristic of canonical NMD substrates is a long 3′-untranslated region (3′-UTR). Long 3′-UTRs delay translation termination by keeping terminating ribosomes away from termination-stimulating factors, such as the poly(A)-binding protein (PABP), which binds to the poly(A) tail of mRNAs [1,2,3,4]. Another characteristic of canonical NMD substrates in mammalian cells is the presence of at least one exon-junction complex (EJC) downstream of the PTC [5,6,7]. During splicing, EJCs assemble 20–24 nucleotides (nt) upstream of exon-exon junctions [8]. As normal stop codons are typically situated in the last exon of a mRNA, EJCs are dissociated from transcripts during translation, resulting in EJC-free mRNAs [9]. When exon-exon junctions are situated more than 50–55 nt downstream of PTCs, the remaining EJCs give rise to abnormal translation termination and activate the NMD machinery by recruiting NMD factors to the terminating ribosomes [10,11,12].

NMD is also involved in the control of gene expression and targets ~10% of cellular transcripts [13,14]. Thereby, NMD takes part in a variety of cellular mechanisms, such as the regulation of the cell cycle, cell viability, DNA damage response and innate immune response to viral infections [13,14,15,16,17]. Moreover, dysregulation of the NMD pathway causes serious pathologies, such as cellular stress and cancer, and is associated with neurodevelopmental disorders (NDDs) [17,18,19]. Hence, elucidating the molecular mechanisms governing NMD is essential for the development of novel therapeutic strategies.

In this review, we describe the different mammalian NMD pathways identified to date, as well as the main NMD factors—the up-frameshift proteins (UPFs). Furthermore, we address the implication of NMD in human pathologies, including NDDs, cancer and viral infections.

## 2. Mammalian NMD Pathways

**Figure 1:**
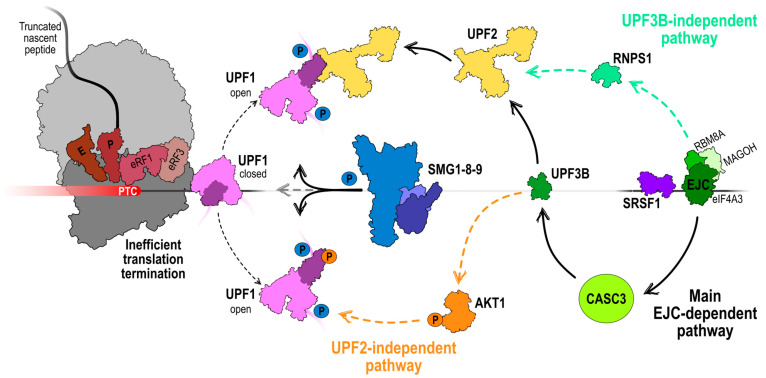
Scheme showing different factor requirements for the main and alternative pathways leading to the activation of the key NMD factor UPF1. Upon inefficient translation termination at a premature termination codon (PTC), NMD is triggered by two-step activation of UPF1: transition from its closed to its open conformation, and hyper-phosphorylation of its N- and C-termini by SMG1-8-9 kinase. In the canonical EJC-dependent NMD pathway (black arrows), CASC3, UPF3B and UPF2 are required for UPF1 activation. UPF2 promotes the change in UPF1’s conformation by directly interacting with its CH domain. In the CASC3- and UPF3B-independent NMD pathway (dashed aquamarine arrows), RNPS1 directly recruits UPF2 to activate UPF1. In the UPF2-independent NMD pathway (dashed orange arrows), CASC3, UPF3B and AKT1 are required for the activation of UPF1. AKT1 promotes the change in UPF1’s conformation by phosphorylating its CH domain. SRSF1 enhances all NMD pathways by interacting with the EJC and NMD factors.

In mammals, NMD targets a wide range of RNAs, from mRNAs harbouring PTCs due to mutations, to mRNAs with long 3′-UTRs or short upstream open reading frames (uORFs) in their 5′-UTRs, as well as selenoprotein-encoding mRNAs, but also small nucleolar RNAs (snoRNAs) and long non-coding RNAs (lncRNAs) [20,21,22,23,24,25,26,27]. NMD depends on translation of the RNA transcript, with NMD functions spanning from translational quality control to gene regulation. Accordingly, mammalian NMD is tightly regulated. In fact, different NMD pathways were identified, each requiring distinct combinations of NMD factors [13,14]. All NMD pathways have in common that they involve UPF1, which, therefore, is considered as the key NMD factor (Figure 1) (reviewed in [14,28,29]).

In the canonical model of NMD, a PTC is defined by the presence of one or several EJCs associated with UPF2 and UPF3B in the 3′-UTR of the mRNA. In this model, translation termination at the PTC is aberrant and slow, allowing the assembly of the SURF surveillance complex on the terminating ribosome. SURF consists of the SMG1-8-9 kinase complex, UPF1 and the eukaryotic release factors eRF1 and eRF3 [30]. UPF1, as part of the ribosome-bound SURF complex, can interact with EJC-associated UPF2 and UPF3B, which gives rise to a so-called decay-inducing complex (DECID) [30,31]. Formation of the DECID complex leads to activation of UPF1 by SMG1-8-9-mediated hyper-phosphorylation [32]. Hyper-phosphorylated UPF1 triggers a series of events, ultimately leading to mRNA degradation which is suggested to take place in processing bodies (P-bodies) [33]. Notably, hyper-phosphorylated UPF1 promotes post-termination ribosome recycling. This step depends on UPF1’s ATP hydrolysis activity, which is activated by the UPF1-UPF2 interaction [34,35]. Hyper-phosphorylated UPF1 also binds eukaryotic translation initiation factor 3 (eIF3), which is required for ribosome recycling and translation re-initiation [36,37]. UPF1 binding to eIF3 is suggested to prevent new rounds of cap-dependent translation on nonsense mRNA [38]. Importantly, hyper-phosphorylated UPF1 is a scaffold for the recruitment of mRNA decay initiators SMG6 and SMG5-7 [39]. SMG6 endonuclease is activated by interaction with UPF1, and SMG5-7 and was shown to cleave mRNA close to the PTC [25,40,41]. SMG5-7 recruits additional factors for mRNA decapping (DCP2) and deadenylation (CCR4/NOT) [42,43]. Ultimate degradation of the unprotected mRNA is subsequently completed by exonuclease XRN1 and the exosome complex [43]. SMG5-7 also recruits the protein phosphatase 2A (PP2A) responsible for UPF1 dephosphorylation. Dephosphorylation of UPF1 results in the release of SMG6 and SMG5–7, and allows the recycling of UPF1 for novel rounds of NMD [33,44].

This canonical model is also referred to as EJC-dependent NMD (Figure 1). In fact, the presence of an EJC downstream of the stop codon is the strongest marker of NMD substrates [7]. More recently, variations of this NMD model have been described, varying in EJC composition. The core of the EJC is formed by eIF4A3, the protein mago nashi homolog (MAGOH), and the RNA-binding protein 8A (RBM8A, also known as Y14) [45,46,47]. Additional subunits can associate with the EJC core in a mutually exclusive manner. The cancer susceptibility candidate gene 3 (CASC3) protein (also known as BTZ or MLN51) is associated to the EJC in canonical NMD, and it has also been shown that CASC3-bound EJCs can mediate NMD, independently of UPF2 [47,48,49,50]. In this UPF2-independent ‘branch’ of EJC-dependent NMD (Figure 1), CASC3 is suggested to directly interact with eIF4A3, UPF3B as well as UPF1, with UPF3B binding to UPF1 and eRF3, thereby stabilising UPF1 in the vicinity of the EJC, despite of the lack of UPF2 [48,49,50]. In the canonical NMD pathway, UPF2 promotes UPF1’s ATPase and helicase activities and, thereby, supports NMD activation [51,52,53]. Recent studies have shed light on the mechanisms compensating for the absence of UPF2 in UPF2-independent NMD, revealing the involvement of serine/threonine-protein kinases AKT (Figure 1) [54,55]. AKT1 was first identified as a member of the NMD pathway in a screen of kinase inhibitors for putative NMD modulators [54]. In the same study, co-immunoprecipitation assays showed that AKT1 interacts with UPF1 and UPF3B, but not with UPF2, and that the interaction between AKT1 and UPF1 is dependent on UPF3B [54]. Subsequently, a genetic screen for NMD effectors demonstrated that AKT replaces UPF2 in CASC3-EJCs and is able to activate UPF1 [55].

UPF3B-independent NMD presents another branch of EJC-dependent NMD, relying on RNPS1 bound to EJCs (Figure 1) [48]. RNSP1-EJCs recruit UPF2 in the absence of UPF3B. It was shown that RNPS1-bound EJCs and CASC3-bound EJCs have distinct subcellular localisations and lifetimes [50]. RNPS1 associates to EJCs during co-transcriptional splicing. Consistently, RNPS1-EJCs are mostly found in the nucleus and in the cytoplasm shortly after mRNA export from the nucleus [50,56]. Therefore, RNPS1-enhanced NMD is suggested to target mRNAs translated in the nuclear periphery immediately after their export [50,56]. In contrast, CASC3 associates with EJCs in the cytoplasm, during a compositional switch from RNPS1 to CASC3 that is either pre- or co-translational. Thus, CASC3-enhanced NMD is suggested to occur during later stages of the mRNA lifetime [49,50].

During co-transcriptional splicing, factors belonging to the serine- and arginine-rich (SR) protein family bind to nascent mRNAs, and their presence is suggested to promote NMD [57,58,59]. In fact, SR Splicing Factors 1 (SRSF1) directly interacts with UPF1, recruiting it to mRNAs in the nucleus. Moreover, co-immunoprecipitation assays indicate that SRSF1 interacts with SMG7 and PP2A, which are both implicated in the dephosphorylation of UPF1 [59,60]. This suggests that SRSF1 could enhance NMD by promoting the recycling of this NMD factor [59,61,62].

The differentiation of NMD into several pathways or branches highlights the importance of NMD as a regulatory mechanism of gene expression in mammalian cells. Several cases of intricate modulation of NMD have been reported for specific cellular contexts. For example, mRNAs translated at the endoplasmic reticulum (ER), or in neuronal dendrites and axons, are targeted by localised NMD [63,64,65]. The ER membrane is the place for translation and translocation of secreted proteins and integral membrane proteins, and the ER is also where the unfolded protein response (UPR) takes place [66,67]. The expression of several UPR factors (e.g., ATF-3, ATF-4, PERK and IRE1α) was shown to be regulated by NMD [68,69]. Notably, the neuroblastoma-amplified sequence protein (NBAS) and the helicase DHX34 were identified as specialised NMD factors, targeting membrane-associated mRNAs and mRNAs encoding proteins of stress response pathways, as well as NMD factor-encoding mRNAs, including UPF1-mRNA [64,69]. The NBAS is suggested to assist in an ER-associated NMD pathway by recruiting UPF1 to the ER. The NBAS-mediated NMD is part of a feedback loop that responds to cellular stress, activates the cellular stress response and leads to downregulation of NMD [64]. The integrated stress response inhibits global NMD, mediated by UPF1’s isoform 2, which is most abundant in mammalian cells. This is achieved by translation inhibition via phosphorylation of the α-subunit of eukaryotic initiation factor 2 (eIF2α) [70].

In addition to the distinct factor requirements of the NMD branches, several NMD factors exist in different versions. One example is UPF3B and its paralog UPF3A (see below) [71,72,73,74,75,76]. UPF3B is the main form in most tissues, but UPF3A is most abundant in adult testis [74]. In male germ cells, meiotic sex chromosome inactivation causes transcriptional silencing of the UPF3B gene, which is located on the chromosome X. This downregulation of UPF3B results in an upregulation of UPF3A and the stabilisation of a specific subset of mRNAs encoding critical genes required for spermatogenesis and male fertility, which would otherwise be degraded by NMD [73,74]. A further example of sophisticated modulation of NMD is the existence of two UPF1 isoforms in mammals: UPF1-1 and UPF1-2 [77,78]. UPF1-2 is the main isoform in human tissues, and it is the main NMD factor in standard cellular conditions [78]. However, UPF1-1 was reported to target mRNAs translated at the ER, e.g., mRNAs encoding integral membrane proteins, even during ER stress when UPF1-2 mediated NMD is downregulated (see above) [78]. Despite down-regulated translation, UPF1-1 was shown to be able to activate NMD in stress conditions. This increased activity was linked to UPF1-1’s prolonged residence time on mRNAs compared to UPF1-2 (discussed below) [78].

## 3. Core NMD Factors

### 3.1. UPF1

**Figure 2:**
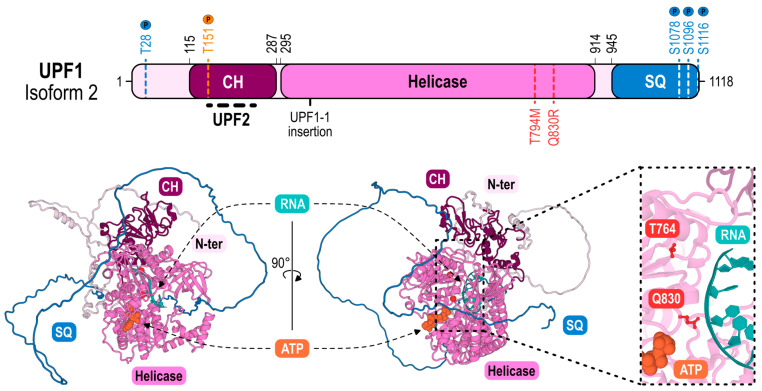
Domain architecture (above) and AlphaFold2 structure prediction (below) of human UPF1 isoform 2. UPF2 interacts with UPF1’s CH domain (thick dashed line). UPF1 is phosphorylated at T28, S1078, S1096 and S1116 (blue) by SMG1, and T151 (orange) by AKT1 kinase. Missense mutations associated with neurodevelopmental disorder are found in the UPF1 helicase domain (red dashed lines in protein scheme; red spheres in the predicted protein structure) [90,91]. The positions of RNA and ATP were modelled by aligning the structure of UPF1’s helicase domain (PDB ID: 2XZO) to the AlphaFold2-predicted UPF1 model [52]. CH, cysteine-histidine-rich; SQ, serine-glutamine-rich.

UPF1 is highly conserved in eukaryotes. It comprises a central helicase domain, flanked by an upstream cysteine-histidine-rich (CH) domain and a downstream serine-glutamine-rich (SQ) domain (Figure 2) [29,51,52,77,79,80]. UPF1’s central role in NMD relies on its capacity to bind RNA, which is supported by its helicase domain. However, the CH domain and the N- and C-terminal regions of UPF1 were also shown to have regulatory functions of UPF1-mediated NMD activation [39,52,55,79].

UPF1 is a superfamily 1 (SF1) helicase, and its RNA-binding, translocase and unwinding activities are linked to ATP-binding and hydrolysis [81,82]. The helicase domain structure of UPF1 is well-characterised [52,80]. A wide range of conformations have been observed in structural studies, shedding light on the molecular dynamics linking UPF1’s RNA-associated catalytic functions to ATP-binding and hydrolysis (reviewed in [29]). ATP binding hinders the binding of RNA by UPF1 [81], and UPF1’s ATPase activity is essential to NMD [38,83,84]. The ATPase activity has been shown to be required for UPF1 to remodel ribonucleoprotein complexes (RNPs) by fuelling its RNA translocase and unwinding activities [34,85,86,87], promoting ribosome recycling at PTCs [34,86], displacing protective RNA-binding proteins [78,85] and disassembling NMD complexes for efficient RNA degradation [83]. A recent study using a range of UPF1 ATPase and helicase mutants has shown that ATP-hydrolysis stimulates dissociation from the RNA in a helicase-decoupled manner [84]. Intriguingly, UPF1 mutants with impaired helicase activity but intact ATPase function still support NMD [84]. In contrast, mutants deficient in the ATPase activity result in compromised NMD substrate selectivity [88,89]. Together, these results suggest that UPF1’s ATPase activity is essential for NMD, due to stimulation of RNA dissociation and concomitant release of non-NMD substrates, while UPF1’s helicase activity, which unwinds RNA and remodels RNPs, is not crucial for NMD [84].

The importance of RNA dissociation and helicase functions of UPF1 for efficient NMD is supported by the existence of different regulatory features within UPF1. Firstly, biochemical studies show that the CH domain enhances RNA-clamping and reduces the RNA-unwinding activity of UPF1 [92]. This is supported by a structure of yeast Upf1, showing that the CH domain associates with the helicase domain in a closed conformation, forming a longer RNA-binding channel [52]. In the context of NMD, UPF2-binding to UPF1’s CH domain removes auto-inhibition of UPF1 helicase and of RNA dissociation. Structural data of UPF1, in complex with the C-terminus of UPF2, shows that the CH domain of UPF1 is displaced upon UPF2-binding into an open conformation [52,79], resulting in decreased RNA-clamping and increased RNA-unwinding by UPF1 [51,52]. Recent biochemical and biophysical studies have shown that binding of UPF2 to UPF1 directly promotes dissociation of UPF1 from RNA in a non-competitive manner [53]. A low-resolution cryo-EM structure of the EJC, in complex with RNA and the three UPF proteins, shows that UPF1 is found in the vicinity of the RNA 3′-end [93]. Based on this finding, it was suggested that, upon association with the EJC-UPF3B-UPF2 complex, UPF1 is activated and dissociates from the mRNA, but stays anchored to the mRNP via the EJC. More recently, a similar activating function was attributed to AKT [55]. In the absence of UPF2, AKT1 cooperates with EJCs containing CASC3 and UPF3B [54,55], and activates UPF1 helicase activity by phosphorylating threonine residue 151 in the CH domain [55]. Earlier structures of UPF1 CH show that T151 (T90 in yeast) is located at the interface between the CH and the helicase domains, where RNA is bound [52,79]. Accordingly, phosphorylation of T151 could lead to the repulsion of the CH domain from the RNA, inducing an open conformation [55]. Taken together, UPF2-binding and AKT1 phosphorylation of the CH domain are both suggested to induce a switch from a closed to an open conformation. This switch reduces the RNA-binding and increases helicase activities of UPF1, triggering NMD activation.

Besides the CH domain, RNA dissociation and helicase functions of UPF1 are suggested to be regulated by a loop located within its helicase domain. In the most abundant isoform of UPF1 (UPF1-2), this loop is 11-residues long. The alternatively spliced UPF1-1 isoform has an 11-residues insertion, resulting in a 22-residues long loop (Figure 2) [77]. Structural and biochemical data show that this loop is part of the RNA-binding channel and directly affects RNA-binding: while the shorter loop of UPF1-2 is oriented towards the helicase core and competes with RNA-binding, the longer loop of UPF1-1 is more flexible and oriented towards the solvent, thus not interfering with RNA-binding [52,77,80,94]. As a result, UPF1-1 showed increased RNA-binding in the presence of ATP, along with increased ATP hydrolysis in the presence of RNA, in comparison to the more abundant UPF1-2 isoform [77]. Consistently, RNA-unwinding and translocase activities were shown to be higher for UPF1-1 than for UPF1-2 [77]. In accordance with these differences in biochemical properties, recent biochemical and transcriptome-wide studies suggest distinct roles for the two UPF1 isoforms [78]. Proteins binding to mRNAs with long 3′-UTRs, such as the polypyrimidine tract-binding protein-1 (PTBP1) or the heterogeneous nuclear ribonucleoprotein L (hnRNP L), protect these mRNAs from NMD mediated by UPF1-2, by inhibiting UPF1-2′s translocase activity and promoting its dissociation from the mRNA [95,96,97]. However, due to the stronger translocase activity of UPF1-1, this isoform can displace protective RNA-binding proteins, such as PTBP1, and thereby promote NMD of mRNAs with very long 3′-UTRs [78], resulting in different target-specificities of the two UPF1 isoforms.

Phosphorylation of UPF1 is the decisive event triggering NMD, allowing it to proceed to the mRNA degradation step after its recognition by the NMD machinery. UPF1 is hyper-phosphorylated by the SMG1–8–9 kinase complex at serine/threonine-glutamine motifs located in its disordered N- and C-terminal regions (e.g., T28, S1078, S1096, S1116, Figure 2) [32,98]. Several of these phosphorylation sites share a consensus leucine-serine-glutamine (LSQ) sequence [99]. The structural study of the SMG1–8–9 kinase complex with a peptide mimicking UPF1 residues 1074–1084 (part of the SQ domain) reveals that these LSQ motifs bind within a hydrophobic cage in the vicinity of the SMG1 kinase active site [29,100]. These data highlight the importance of UPF1’s LSQ motifs for optimal phosphorylation specificity and efficiency of the SMG1–8–9 kinase complex. Hyper-phosphorylated UPF1 provides a platform to recruit SMG6 endonuclease and SMG5-SMG7 to the NMD substrate, initiating mRNA decay [32,39,44,88,101,102].

Despite the vital role of UPF1 in NMD, the UPF1 enzymatic functions specifically implicated in NMD are still enigmatic. UPF1’s CH domain is a good illustration of UPF1’s functions, which remain poorly understood. Structural studies of the CH domain of UPF1 showed a unique association of two RING-box-like modules [29,52,79], which are characteristic of some families of E3 ubiquitin ligase enzymes. Interestingly, biochemical studies showed that yeast Upf1 self-ubiquitinates when Upf3 and the E2 ubiquitin-conjugating enzyme Ubc3 are present [103]. In human cells, the transcription factor MYOD was shown to be down-regulated by UPF1 as a result of MYOD ubiquitination and proteasomal degradation rather than decay of MYOD-encoding mRNA [104], indicating that UPF1’s E3 ligase function is separate from the NMD function in this context. Further investigation into UPF1’s E3 ubiquitin ligase activity is required to identify the E2 ubiquitin conjugating partner for UPF1 and its substrate specificity in human cells. In fact, little is known about the fate of truncated and possibly misfolded proteins produced from NMD-targeted mRNAs. Therefore, it is tempting to speculate about a role for UPF1 in the ubiquitination and subsequent degradation of nonsense mRNA-encoded polypeptides. A study using normal and nonsense reporter mRNAs encoding the same protein showed a proteasome-dependent destabilisation of the newly synthesised polypeptide encoded by nonsense mRNA [105]. The role of the proteasome in NMD-related degradation of nonsense polypeptides is further supported by recent assays using a double-reporter mRNA allowing the decoupling of mRNA decay and protein quality-control in NMD [106]. In no-go and non-stop mRNA decay, the associated ribosome-associated quality-control (RQC) pathway involves E3 ubiquitin ligase LTN1/Listerin for nascent chain ubiquitination and subsequent proteasomal degradation [107,108]. In contrast, a recent study suggests that NMD-associated protein degradation does not require LTN1 or UPF1 as E3 ubiquitin ligases but involves the CCR4–NOT transcription complex subunit 4 (CNOT4). CNOT4 was identified in genome knockdown and knockout screens as being responsible for the ubiquitination signal leading to proteasomal degradation of the nonsense polypeptide [106]. However, the molecular mechanisms linking mRNA decay and nonsense polypeptide ubiquitination in NMD remain to be elucidated.

### 3.2. UPF2

**Figure 3:**
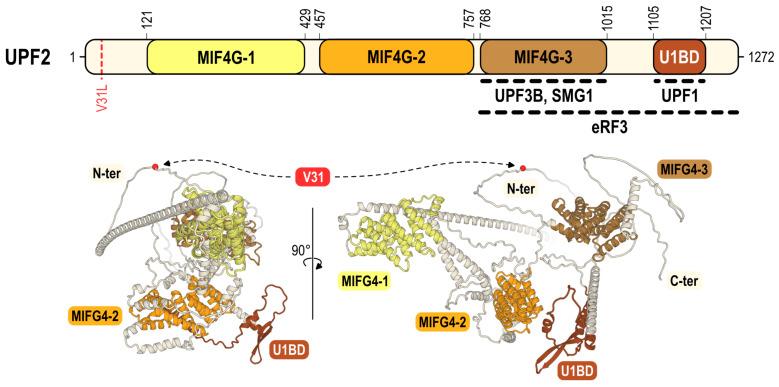
Domain architecture (above) and AlphaFold2 structure prediction (below) of human UPF2. UPF2 interaction partners are indicated by thick and dashed lines. V31L in UPF2 was found to be associated with neurodevelopmental disorder (red dashed line in protein scheme and red sphere in predicted UPF2 structure) [91,115]. MIF4G, middle portion of eIF4G; U1BD, UPF1 binding domain.

UPF2 associates with the EJC in nucleoplasmic and cytoplasmic fractions of human cells, and is mostly found in the cytoplasmic perinuclear region [109]. UPF2 is composed of three tandem middle portions of eIF4G (MIF4G) domains (Figure 3) [110], which are suggested to form a ring-shaped structure [111]. The first two MIF4G domains (MIF4G-1 and MIF4G-2) are conserved from yeast to human but have an unknown function. The third MIF4G (MIF4G-3) domain plays a key role in mediating the assembly of the NMD machinery: MIF4G-3 interacts with SMG1 kinase and UPF3B and helps to activate SMG1 kinase activity [30,110]. In the complex with SMG1, UPF2′s MIF4G-3 and its C-terminal part were phosphorylated by SMG1 kinase, but the significance of this step remained elusive [110]. Furthermore, UPF2 is suggested to support dissociation of hyper-phosphorylated UPF1 from the SMG1-8-9 kinase complex [98].

UPF2′s C-terminal UPF1-binding domain (U1BD) (Figure 3) is natively disordered in free UPF2 protein, as determined in NMR studies [51]. In the presence of UPF1, UPF2′s C-terminal domain folds into an alpha-helical and a beta-hairpin structure which binds UPF1’s CH domain [51]. This U1BD-CH interaction induces an open UPF1 CH-helicase conformation with lower affinity for RNA and increased UPF1’s ATPase activity (see above) [51].

In a recent study, UPF1-UPF2 complexes were shown to have a low RNA affinity, despite the fact that both NMD factors have been shown to interact with RNA [53]. The authors performed fluorescence anisotropy experiments and reported affinities in the low nanomolar range for UPF2 MIF4G domains 1-2, as well as MIF4G domain 3 with the U1BD [53]. This corroborates previous work showing that UPF2′s MIF4G domain 3 binds RNA in electrophoretic mobility shift assays (EMSAs) via conserved basic and aromatic residues [112] and work showing ribosome and RNA binding of UPF2 in complex with UPF3B [113,114]. In complex with UPF1, UPF2 was shown to help UPF1-RNA dissociation, likely by binding to a region allosteric of UPF1’s RNA-binding site [53].

UPF2 was also shown to interact with eRF3 via its MIF4G-3 domain and C-terminal region [111]. However, UPF2 did not have an impact on the efficiency of translation termination in a reconstituted translation assay [113]. Therefore, this interaction is unlikely to contribute to the aberrant and slow translation termination at a PTC on an NMD substrate.

### 3.3. UPF3A and UPF3B Paralogs

**Figure 4:**
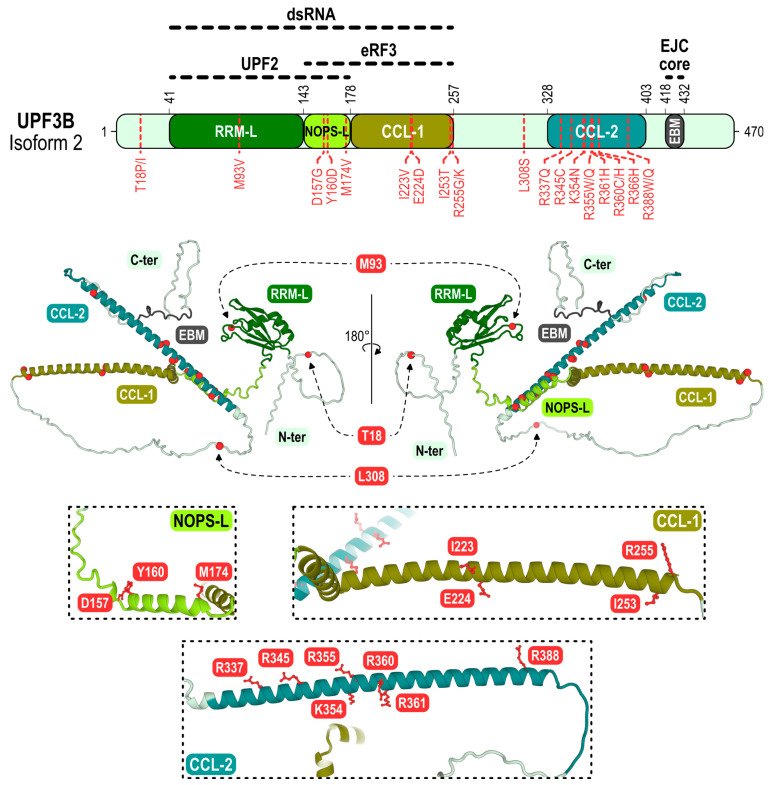
Domain architecture (above) and AlphaFold2 structure prediction (below) of human UPF3B isoform 2. UPF3B interaction regions with other proteins are indicated by thick and dashed lines. Missense mutations related to neurodevelopmental disorders are highlighted by red dashed lines in UPF3B protein scheme and red spheres in predicted UPF3B structure [90,91]. RRM-L, RNA recognition motif-like domain; NOPS-L, NONA/paraspeckle-like region; CCL, coiled-coil-like region; EBM, EJC-binding motif.

UPF3B is also called UPF3X, as it is encoded by the X chromosome. The transcription of the UPF3B gene is activated by the SATB2 transcription regulator by direct binding to the UPF3B gene promoter [116]. UPF3B is a nucleocytoplasmic shuttling protein which binds to the EJC in the nucleus and is then exported to the cytoplasm in complex with the EJC [5]. Human UPF3B comprises an RNA recognition motif-like (RRM-L) domain in its N-terminal, followed by a NONA/paraspeckle-like (NOPS-L) region, two predicted coiled-coil-like (CCL-1 and CCL-2) regions and a C-terminal EJC-binding motif (EBM) (Figure 4) [114].

UPF3B interacts with UPF2′s MIF4G-3 through its RRM-L domain and NOPS-L region [114]. The crystal structure of the complex revealed an intimate interaction where UPF2′s MIF4G-3 domain wedges between the RRM-L and NOPS-L domains of UPF3B. The NOPS-L-mediated interaction with UPF2′s MIF4G-3 is essential for high-affinity binding [112,114]. In fact, the UPF3B Y160D mutation causing neurodevelopmental disease is located in the UPF3B NOPS-L region. On the molecular level, aspartate residue 160 is displaced from a hydrophobic cleft formed by UPF2′s MIF4G-3, causing a ~40-fold decrease in UPF2-UPF3B interaction affinity [114]. This weakened affinity between UPF3B and UPF2 was shown to lead to an upregulation of UPF3A (see below) [117] and decreased NMD efficiency, as evidenced by increased mRNA levels of factors regulating neurodevelopment, such as ATF4 and ARHGAP24 [65]. UPF3B was also found to weakly interact with UPF1 in the absence of UPF2, which may be important in UPF2-independent NMD where UPF1-UPF3B complex formation may be stabilised by additional factors (Figure 1) [113].

More recently, UPF3B was shown to interact with single- and double-stranded RNA (ssRNA/dsRNA), as well as DNA, with a preference for dsRNA [114]. This agrees with a previous report showing that UPF3B interacts with 80S ribosomes which have dsRNA expansion segments [113]. UPF3B’s RRM-L, NOPS-L and the CCL-1 regions are required for high-affinity RNA interaction (Figure 4). Interestingly, an RNA-induced oligomerisation of UPF3B was observed which is reminiscent of paraspeckle protein behaviour [114]. The UPF3B-ribosome interaction agrees with the finding that UPF3B is enriched in the nucleolus where ribosomes are assembled in human cells [65]. A reconstituted mammalian translation system was used to show that UPF3B impacts the efficiency of translation termination [113]. UPF3B binds eRF1 and eRF3 and delays stop codon recognition and subsequent peptide release, slowing down translation termination [113]. After peptide release, UPF3B promotes the dissociation of post-termination 80S ribosomal complexes, releasing the bound mRNA [113].

The EBM of UPF3B (Figure 4) interacts with the EJC core composed of eIF4A3, RBM8A and MAGOH [92,118]. Mutation of residue R423 to alanine in the EBM inhibits UPF3B’s interaction with RBM8A and leads to decreased NMD efficiency, similar to a UPF3B EBM deletion mutation (UPF3B△421-434) [118,119].

UPF3A is a paralog of UPF3B evolutionary conserved in vertebrates [74]. In human cells, two UPF3A isoforms (UPF3AL and UPF3AS) were identified, caused by alternative splicing in exon 4 [71]. The longer isoform UPF3AL retains the exon 4, which encodes the β5 strand of the RRM-L domain and the NOPS-L region, and therefore can interact with UPF2. In contrast, UPF3AS lacks exon 4 and, thus, loses important residues in NOPS-L for UPF2 interaction [114]. UPF3A (UPF3AL) and UPF3B directly compete for binding UPF2 by binding to the same sites in MIF4G-3 [114]. Surprisingly, UPF2′s MIF4G-3 binds UPF3AL with ~10-fold higher affinity than UPF3B due to additional interactions between UPF3AL’s NOPS-L region and UPF2′s MIF4G-3 domain [114]. However, expression levels of UPF3B are significantly higher than UPF3A in most mammalian cells. Therefore, the downregulation of UPF3A in the presence of UPF3B is likely achieved because UPF3B can outcompete UPF3A for UPF2-binding, thereby negatively affecting UPF3A’s protein half-life [72]. The ‘free’ UPF3A (not stabilised by UPF2) was shown to be quickly degraded in cells [72]. Consistently, UPF2 overexpression leads to UPF3A stabilisation, even in the presence of UPF3B [72]. Vice versa, UPF2 knockdown prevents UPF3A stabilisation, even if the UPF3B expression is also inhibited [72].

Previously, UPF3A was described as a weak NMD factor or an NMD inhibitor [71,74]. UPF3A was suggested to interfere with the NMD machinery by sequestering UPF2, likely due to its weaker affinity for the EJC in comparison to UPF3B. Consistently, when replacing the human UPF3A’s EBM with UPF3B’s EBM, the mutated UPF3A turned from an NMD repressor into an NMD activator. Similarly, by deleting the EBM region in UPF3B, the mutated UPF3B became an NMD repressor [74]. More recently, UPF3A was shown to rescue NMD activity when UPF3B was knocked out, and NMD inhibition could only be achieved when UPF3A and UPF3B were co-depleted [75,76]. This indicates that UPF3A is an active NMD factor, similar to UPF3B. Taken together, it appears that UPF3A stabilises or destabilises nonsense mRNAs, depending on the specific substate and cellular context. For instance, it was observed that nonsense mRNAs stabilised by UPF3A tend to have a longer 3′-UTR than mRNAs destabilised by UPF3A [74].

## 4. Implication of NMD Factors in Human Disease

### 4.1. Neurodevelopmental Disorders (NDD)

As one of the post-translational regulation pathways in eukaryotic cells, NMD plays an important role in neurodevelopment by regulating gene expression [120]. Approximately 80% of all mRNAs linked to NDD are targeted by NMD [65,121]. During neural differentiation, NMD activity is downregulated. This is achieved partially by targeting mRNAs encoding UPF1 and CASC3 through microRNA-miR-128 [122]. However, despite downregulation of NMD factors, functional NMD is essential for neurodevelopment [65]. In agreement, NMD impairment is found to be associated with schizophrenia, intellectual disorder (ID) and autism spectrum disorder (ASD) [65].

Reduced NMD efficiency can be caused by mutations in UPF2 [123,124] and UPF3B [117,125,126,127,128,129,130,131]. Disease-associated nonsense and frameshift mutations in UPF2 or UPF3B introduce PTCs in their transcripts, such that their mRNAs are targeted by alternative NMD pathways (UFP2-independent NMD or UPF3B-independent NMD) (Figure 1). Resulting reduced levels of NMD factors UPF2 or UPF3B lead to a reduction of the efficacy of the canonical NMD pathway. Missense mutations in UPF3B (Figure 4 and Table 1) reduce the interactions with other NMD factors, including UPF2 [114] and RBM8A [119], leading to inefficient NMD (see above) [65,114,119]. A mutation in UPF2′s N-terminus (Figure 3 and Table 1) has been found in NDD patients (ClinVar accession VCV000996716.1) [90], but the molecular basis of pathogenesis remains to be elucidated. Mice lacking UPF2 in the forebrain were found to have deficits in spatial and contextual long-term memory (LTM) and long-term potentiation (LTP) [124]. This agrees with the learning and memory deficits found in NDD patients [124]. In addition, UPF2-deficient mice exhibited an increased neuroinflammatory response, and treatment with anti-inflammatory drugs was found to reduce brain inflammation and to improve their LTM and LTP [124]. UPF2 single allele deletion is associated with NDDs as well, leading to a more than two-fold transcriptome dysregulation with a very high (95%) similarity to disease-causing mutations in UPF3B [123].

In cell culture experiments, UPF3B missense mutations did not change UPF3B’s cellular location, but caused reduced NMD efficiency and impaired neuronal differentiation [65,120]. For example, ARHGAP24 mRNA, which encodes a GTPase-activating protein and influences neurite outgrowth, as well as neurite branching, is a canonical NMD target. ARHGAP24 mRNA was found to be significantly upregulated in some NDD patients, having mutations in UPF3B [65,117]. Similarly, transcription factor ATF4, known to be required for neuronal development and plasticity, is upregulated in UPF3B-mutated cells [65]. Importantly, in all NDD patients harbouring UPF3B mutations, the upregulation of UPF3A protein levels is correlated with the severity of patients’ disease phenotypes in a reverse way, i.e., less severe disease correlates with higher UPF3A protein levels [117,121].

Two mutations in the UPF1 helicase domain have been found in NDD patients (Figure 2 and Table 1). Homozygous deletion of UPF1 is embryonically lethal in mice, highlighting the importance of UPF1 [134]. Moreover, copy number variants in UPF2, UPF3A, eIF4A3, RBM8A, RNPS1 and SMG6 NMD factors have been identified in NDD patients, associating any imbalance of these NMD factors with NDD aetiologies [123]. Similarly, disease-causing SMG8 mutations are reported to interfere with interactions with SMG9 and/or SMG1, thereby influencing NMD efficiency [135,136].

In addition, hyper-activated NMD was recently reported to be associated with NDD [137]. Loss of the fragile X syndrome protein (FMRP) is a major aetiology of autism and ID [137]. Under normal conditions, FMRP is recruited to NMD substrates by direct interaction with UPF1, repressing NMD. FMRP deficiency increases UPF1 phosphorylation levels and leads to hyper-activated NMD in cells from fragile X syndrome (FXS) patients [137]. Hyper-activated NMD can be partially reversed by small molecules inhibiting NMD, as evidenced by the restoration of early and mature neuron marker expression and of enhanced neurite outgrowth [137].

### 4.2. Cancer

Cancer is assumed to start from abnormal clonal expansion of a single cell. The cancer genome is enriched with somatic point mutations and insertion/deletion (indel) mutations, many of which encode PTC-containing transcripts [138,139]. Analysis of cancer genomes and exomes revealed that PTC-containing tumour-suppressor transcripts are frequently degraded by NMD [7], whereas PTC-containing transcripts of oncogenes frequently encode dominant-negative proteins and evade NMD [7,139]. Accordingly, inhibition of NMD is proposed to benefit cancer treatment by promoting the expression of tumour-suppressor proteins. For example, mutations in the tumour-suppressor gene Tp53 are found in more than 50% of human cancers, and most mutations are nonsense mutations [140]. The function of p53 tumour-suppressor protein is to induce cell apoptosis. NMD inhibition through a combination treatment of NMDI14, which disrupts the UPF1-SMG7 interaction, and a stop codon read-through drug, G418, led to expression of full-length p53 in different cancer cell lines, ultimately leading to cell death (Table 2) [141]. Likewise, NMD inhibition in microsatellite instability colorectal cancer promoted nonsense mRNA expression, including HSP110DE9 which has a dominant negative activity against oncogenesis, and suppresses cell proliferation (Table 2) [139].

Since indel mutations in ORFs often lead to frameshift mutations, and NMD inhibition promotes expression of such proteins with truncated, mutated C-terminal, these new proteins have the potential to be processed into neoantigens [19,142]. Neoantigens derived from these aberrant proteins present tumour cell-specific antigens, enabling the immune system to recognise, attack and destroy the tumour cell [142]. Moreover, NMD inhibition can influence RNA alternative splicing by altering exon usage. This event can also lead to novel neoantigen expression [143]. Using a prostate-specific membrane antigen (PSMA) aptamer-*SMG1* siRNA conjugate, colorectal carcinoma cell growth was significantly depressed in mice, and this therapeutic effect was more than additive when combined with 4-1BB aptamer treatment (Table 2) [142]. Similarly, transfecting B-cell lymphomas with a CD40-agonist aptamer *SMG1*-shRNA chimera inhibited NMD activity, due to SMG1 kinase knockdown, and improved survival of mice with B-cell lymphomas (Table 2) [144]. Moreover, UPF2 knockdown by aptamer-linked siRNA chimeras boosted CD8+ T-cell anti-tumour immunity and inhibited murine breast tumour growth (Table 2) [143]. Interestingly, in cancer cells having mutations in U2AF1 or SF3B1, two components of the U2 spliceosome, NMD is found to be attenuated [145]. Further weakening of the NMD activity by SMG1 kinase inhibition led to DNA damage and chromosomal instability in these cancer cells and specifically killed them, providing an alternative therapy for patients [145].

**Table 2:** Effects of NMD inhibition/activation on cancer cells.

	Cancer		Potential Therapeutic Approach			Ref.
	Cancer Type, Cell Line	Characteristics	Strategy	Method	Effect	
**NMD inhibition**	Small-cell lung cancer, cell line N417 Breast cancer, cell line HDQP-I	PTC mutationsin Tp53 gene	**Promoting** **expression** **of tumour** **suppressor** **proteins**	NMDI14 (disruption of UPF1-SMG7 interaction), G418 (PTC read-through)	Restoration of full-length p53 expression, leading to cell death	[141]
	Microsatellite instability colorectal cancer	Overexpression of UPF1, UPF2, SMG1, SMG6 and SMG7		Amlexanox or UPF1 siRNA	Decrease in cell proliferation rate	[139]
	Murine colorectal carcinoma, cell line CT26	n.d. ^a^	**Producing** **tumour-specific** **neoantigens** **that can be** **recognised** **by the human** **immune system**	PSMA ^b^ aptamer-SMG1 siRNA conjugate, 4-1BB aptamer	Suppression of tumour growth by PSMA-Smg1 ± 4-1BB treatment	[142]
	B-cell lymphoma	n.d. ^a^		CD40-agonist aptamer SMG1-shRNA chimera	Tumour infiltration by lymphocytes Improved mice survival	[144]
	Murine breast cancer, cell line 4T1E	n.d. ^a^		EpCAM ^c^ aptamer- UPF2 siRNA chimera	Improved CD8+ T-cell immunity Inhibition of tumour growth	[143]
**NMD activation**	Prostate cancer, cell line PC3 Colon cancer, cell line HCT116 Melanoma, cell line A375	eIF2α phosphorylation	**Downregulating** **tumorigenesis** **transcripts**	UPF1 overexpression	Inhibition of tumour growth	[146]
	Human gastric tumour	Inhibition of UPF1 expression			Suppression of cell cycle progression and proliferation Promotion of cell apoptosis Inhibition of EMT ^d^ and metastasis	[147]

^a^ n.d., not defined. ^b^ PSMA, prostate-specific membrane antigen, introduced by exogenous transduction of PSMA expression vector into the cell. ^c^ EpCAM, epithelial cell adhesion molecule. ^d^ EMT, epithelial-mesenchymal transition.

However, in other cases, enhancement of NMD might be beneficial to cancer treatment. In breast cancer, prostate cancer and melanoma, for instance, eIF2α is phosphorylated because of nutrient deprivation, hypoxia and other cellular stresses [146]. NMD efficiency is therefore suppressed, leading to upregulation of many transcripts, some involved in tumorigenesis [146]. Reactivation of NMD by overexpressing UPF1 inhibited tumour growth, as shown in prostate cancer, colon cancer and melanoma cell lines, but not in all cell lines tested (Table 2) [146]. Moreover, UPF1 overexpression suppresses cell proliferation and progression, promotes cell apoptosis, and inhibits cell epithelial-mesenchymal transition (EMT) and metastasis in gastric tumours (Table 2) [147].

Recent bioinformatic studies suggest than some of the NMD factors can be used as prognostic markers for cancers. For instance, UPF3B and SMG5 are highly expressed in hepatocellular carcinoma (HCC) tissue and are associated with poor prognosis in these patients [148,149]. Similarly, UPF3B expression was highly upregulated in colorectal cancer patients at late clinical stages [150].

### 4.3. Viral Infections

Viruses have evolved to maximise the coding capacity of their genomes. Viral evolutionary strategies include alternative splicing of viral transcripts or polycistronic genomes, resulting in viral RNAs with stop codons upstream of EJCs or very long 3′-UTRs, both features that trigger NMD [14]. In this context, NMD acts as a cellular antiviral defence mechanism. In response, viruses have developed different mechanisms to avoid or inhibit NMD. The distinctive characteristic of positive-sense single-stranded RNA (+ssRNA) viruses is the presence of membrane-bound replication factories, from which several protein synthesis factors are excluded, and that were demonstrated to protect viral RNA from RNases [151]. These replication factories are suggested to also exclude NMD factors [151]. Riboviria viruses have developed different mechanisms to avoid NMD, relying on inherent RNA features, making its genome NMD-resistant (cis-strategies), and viral proteins that inhibit NMD (trans-strategies; reviewed in [152]). Interestingly, NMD restriction of viral replication and *vice versa* mechanisms, by which viruses circumvent NMD, have been described mainly for Riboviria viruses, including +ssRNA viruses, a few retroviruses and double-stranded RNA (dsRNA) viruses, but also for a double-stranded DNA (dsDNA) virus (Table 3, Table 4 and Table 5, reviewed in [153,154]).

#### 4.3.1. NMD Restriction of Viral Replication

To date, most viruses that have been identified as being sensitive to NMD are +ssRNA viruses (Table 3). This could be explained by the fact that the replication of +ssRNA viruses takes place solely in the cytosol, with their genomic RNA (gRNA) being translated into non-structural viral proteins directly after entry into the host cell. As some +ssRNA virus gRNAs are polycistronic, they act as mRNAs with long 3′-UTRs and are thus targeted by the NMD machinery. NMD was first found to inhibit viral infection when increased infectivity of Sindbis virus (SINV)-like particles was observed in a transgenic Drosophila strain, where NMD was abrogated by co-expression of a dominant negative UPF1 mutant [155]. Another study using a genome-wide siRNA screen in HeLa cells showed that Semliki forest virus (SFV) viral genome replication is suppressed early in infection, with involvement of UPF1, SMG5 and SMG7, but not SMG6 [156]. Shortening of the SFV gRNA 3′-UTR did not impact the sensitivity of viral replication to UPF1 presence/depletion, suggesting that the 3′-UTR length might not be the only NMD-triggering feature of the RNA [156]. NMD inhibition in mouse fibroblasts transfected with mouse hepatitis virus (MHV) gRNA resulted in higher virus titres, demonstrating that MHV replication is also targeted by the NMD pathway [157]. Additionally, quantitative reverse-transcription–polymerase chain reaction (qRT-PCR) analyses showed that NMD targets MHV gRNA and subgenomic RNAs (sgRNAs) with long 3’-UTRs early in infection [157]. A similar infection restriction by NMD-mediated degradation of viral RNA was observed for Zika virus (ZIKV), using UPF1-depleted human neural progenitor cells (NPCs) [158].

In the case of human immunodeficiency virus-1 (HIV-1), several knockdown studies in human cell lines showed that UPF2 and SMG6 are detrimental to viral protein expression and RNA replication, as they promote viral RNA decay [159,160,161,162]. In contrast, UPF1 knockdown results in impaired viral RNA expression [159,160,163] suggesting that UPF1 is a positive regulator of HIV-1 replication, with roles promoting reverse-transcription and viral RNA export (see below). These functions are probably independent of UPF1’s NMD function [159,160,163].

Kaposi’s sarcoma-associated herpesvirus (KSHV) is a dsDNA virus whose replication is restricted by NMD [164]. The KSHV transcriptome includes numerous potential NMD targets with long 3′-UTRs or intron-containing 3′-UTRs. In agreement, UPF1 or UPF3B depletion was shown to enhance viral gene expression and virion production of KSHV [164]. Formaldehyde crosslinking RNA immunoprecipitation (fRIP)-coupled to high-throughput sequencing identified ORF48 and ORF50 transcripts as being enriched in phospho-UPF1 and targeted by NMD [164]. ORF50 mRNA encodes a transcription factor required for KSHV reactivation. Interestingly, NMD also specifically targets the spliced RNA encoding the active form of the X-box binding protein 1 (XBP1) [164]. XBP1 is a transcription factor activated during the unfolded protein response (UPR), which transactivates the promoter of ORF50 [165,166]. Taken together, NMD is suggested to restrict the reactivation of KSHV by regulating ORF50 mRNA levels, both at transcription and post-transcription levels.

**Table 3:** Sensitivity of animal viruses to NMD factors.

Virus Classification					Effect of NMD Factors			
Name ^a^	Realm	Genome ^b^	Family	Genus	Anti-Viral	None	Pro-Viral	Ref.
MHV	Riboviria	+ssRNA	Coronaviridae	Betacoronavirus	UPF1	n.d. ^c^	n.d. ^c^	[157]
					UPF2			
					SMG5			
					SMG6			
SFV	Riboviria	+ssRNA	Togaviridae	Alphavirus	UPF1	SMG6	n.d. ^c^	[156]
					SMG5			
					SMG7			
SINV	Riboviria	+ssRNA	Togaviridae	Alphavirus	UPF1	n.d. ^c^	n.d. ^c^	[155]
ZIKV	Riboviria	+ssRNA	Flaviviridae	Flavivirus	UPF1	n.d. ^c^	n.d. ^c^	[158]
UUKV	Riboviria	-ssRNA	Phenuiviridae	Phlebovirus	n.d. ^c^	UPF1	n.d. ^c^	[167]
HRSV	Riboviria	-ssRNA	Pneumoviridae	Orthopneumovirus	n.d. ^c^	UPF1	n.d. ^c^	[156]
HIV-1	Riboviria	ssRNA-RT	Retroviridae	Lentivirus	UPF2	n.d. ^c^	UPF1 ^d^	[159,160,161,162,163]
					SMG6			
KSHV	Duplodnaviria	dsDNA	Herpesviridae	Rhadinovirus	UPF1	n.d. ^c^	n.d. ^c^	[164]
					UPF3B			

^a^ Abbreviated virus names: MHV, mouse hepatitis virus; SFV, Semliki forest virus; SINV, Sindbis virus; ZIKV, Zika virus; UUKV, Uukuniemi virus; HRSV, human respiratory syncytial virus; HIV-1, human immunodeficiency virus-1; KSHV, Kaposi’s sarcoma-associated herpesvirus. ^b^ Abbreviated genomic groups: +ssRNA, positive-sense single-stranded RNA; -ssRNA, negative-sense single-stranded RNA; ssRNA-RT, single-stranded RNA reverse transcribing; dsDNA, double-stranded DNA. ^c^ n.d., no data. ^d^ Pro-viral activity of UPF1 can be due to one or several of its functions, including roles in NMD, reverse transcription and mRNA transport.

#### 4.3.2. Viral NMD Evasion cis-Strategies: NMD Resistance Conferred by Inherent RNA Features

Viruses have evolved mechanisms to overcome NMD of their RNAs (Table 4 and Table 5). Several polycistronic viruses depend on programmed ribosome readthrough or frameshifting to produce their replicase proteins. The Moloney murine leukaemia virus (MoMLV), a member of the Retroviridae family, has a readthrough-enhancing pseudoknot immediately downstream of the *gag* termination codon, allowing the production of the Gag-Pol polyprotein. Consistently, reporter assays showed that placing the MoMLV readthrough-enhancing pseudoknot immediately downstream of the reporter ORF prevents decay of the otherwise NMD-sensitive reporter mRNA [168,169].

Similarly, a hairpin structure from the dsRNA Colorado tick fever virus (CTFV) was shown to stabilise a dual-reporter transcript, due to NMD avoidance [169]. Inherent viral readthrough- and frameshift-enhancing elements lead to UPF1 displacement by ribosomes and thus stabilisation of NMD-sensitive viral polycistronic RNAs [169].

**Table 4:** NMD evasion cis-strategies of animal viruses.

NMD-Resistant RNA Feature	Virus Classification					
	Realm	Genome ^a^	Family	Genus	Name ^b^	Ref.
Programmed ribosome readthrough or frameshifting	Riboviria	ssRNA-RT	Retroviridae	Gammaretrovirus	MoMLV	[168]
	Riboviria	dsRNA	Reoviridae	Coltivirus	CTFV	[169]
Regulated RNA splicing and export	Riboviria	ssRNA-RT	Retroviridae	Lentivirus	HIV-1	[170,171]
3′-UTR RSE bound by PTBP1	Riboviria	ssRNA-RT	Retroviridae	Alpharetrovirus	RSV	[95,172]

^a^ Abbreviated genome groups: ssRNA-RT, single-stranded RNA reverse transcribing; dsRNA, double-stranded RNA. ^b^ Abbreviated virus names: MoMLV, Moloney murine leukemia virus; CTFV, Colorado tick fever virus; HIV-1, human immunodeficiency virus-1; RSV, Rous sarcoma virus.

The ssRNA-RT virus has a complex genome that is integrated to the host cell genome before being transcribed and spliced by the cellular machinery. All retroviruses, including HIV-1, have primary retroviral transcripts characterised by a major 5′ splicing site in their 5′-UTR, upstream of the gag gene, and a 3′ splicing site downstream of the pol gene, turning gag-pol into an intron [173]. The HIV-1 genome can produce >30 different mRNAs, with 3 types of splicing patterns: unspliced, once-spliced, and completely spliced [174,175]. The first genes to be expressed during HIV-1 replication (tat, rev and nef) arise from completely spliced mRNAs that are not vulnerable to NMD because they contain no EJC downstream of the termination codon [171]. Subsequently, the transactivating Rev protein binds to incompletely spliced HIV-1 RNAs and mediates their export to the cytoplasm, where they are translated with no susceptibility to NMD [170,176]. During this process, Rev is suggested to interact with UPF1’s CH domain [160]. Immunoprecipitation assays indicate a competition between Rev and UPF2 for UPF1 binding suggesting that UPF2 is excluded from HIV RNPs while UPF1 is present [159,160]. Additionally, overexpression of UPF2 appears to prevent the export of gRNA from the nucleus [159,160].

Some viruses have evolved internal RNA sequences rendering them NMD-resistant: The Rous sarcoma virus (RSV) comprises the RNA stability element (RSE) [95,172,177,178]. The RSV RSE is a 400-nt long region, with a 150-nt minimal functional element, containing a ~30-nt AU-rich stretch at its 5′ end associated to several stem-loop structures [172,177,178]. The RSE is located immediately downstream of the gag termination codon in RSV unspliced RNA, which can serve both as a gRNA and RNA template for the synthesis of Gag and Gag-Pol polyproteins. Translation of only the gag gene results in a 7-kb long 3′-UTR that would normally be recognised by the NMD machinery [172,177,178]. However, RSV RSE was shown to recruit PTBP1, and thereby exclude UPF1 from RSV unspliced RNAs, preventing NMD (see above) [95].

#### 4.3.3. Viral NMD Evasion trans-Strategies: NMD Inhibition by Viral Proteins

In addition to viral RNA features that prevent NMD, viruses can avoid NMD by interacting directly with NMD factors. This results in global NMD inhibition and concomitant upregulation of cellular NMD targets. In the case of coronaviruses (CoV), several studies support an NMD-antagonist role of the nucleocapsid (N) protein (Table 5) [157,179,180]. In agreement, UPF1 was found to interact with the N protein in interactome-mapping studies of the avian infectious bronchitis virus (IBV) and severe acute respiratory syndrome CoV 2 (SARS-CoV-2) [179,180]. However, the molecular mechanism of UPF1 inhibition by the N-protein remains to be elucidated.

With regard to Alphaviruses, UPF1 depletion increased the half-life of viral RNAs in HeLa cells infected with a SFV variant [156]. Similarly, UPF1 suppression resulted in higher virus release from HeLa cells infected with a SFV mutant [156]. It was suggested that SFV replication machinery could inhibit NMD by displacing UPF1 from the positive-sense gRNA [156]. A recent virus-host protein interactome investigation of SFV in HeLa cells identified UPF1 as an antiviral factor interacting with SFV capsid protein [181]. Consistently, expression of the capsid protein induced increased levels of NMD-target RNAs, indicating that it interferes with cellular NMD [181]. However, the molecular mechanism of UPF1 inhibition by SFV’s capsid protein is still enigmatic.

In Flaviviridae, RNAs normally targeted by NMD were shown to be stabilised during infection for hepatitis C virus (HCV), West Nile virus (WNV), dengue virus (DENV) and ZIKV [158,182,183]. Immunoprecipitation experiments in HCV-infected cells showed that this capsid protein interacts with PYM1 (Partner of Y14 and MAGOH homologue 1) [182]. PYM1 is known to interact with both RBM8A (also known as Y14) and MAGOH, and to recycle RNA-bound EJCs [9,184,185]. PYM1 suppression in hepatoma cells leads to less HCV replication, suggesting that PYM1 is a pro-viral factor of HCV [182]. Immunoprecipitation experiments in HCV-infected hepatoma cells indicated a reduced interaction of PYM1 with RBM8A and MAGOH, suggesting that the capsid protein of HCV might interfere with this interaction. Similarly, PYM1 was found to interact with the capsid proteins from DENV, WNV and ZIKV, suggesting that this interaction is conserved among Flaviviridae family members [158,182,183]. Intriguingly and in contrast to HCV, PYM1, along with MAGOH and UPF1, was identified as an anti-viral factor of WNV, DENV and ZIKV (who are from a different Flaviviridae genus than HCV) because knockdown of PYM1 increased viral infection and/or RNA levels in HEKs cells [183]. Fractionation experiments suggest that, by sequestrating PYM1, the capsid protein of Flaviviruses alters the location of RBM8A and MAGOH. Cross-linking immunoprecipitation (CLIP) in WNV-infected cells shows a specific interaction between RBM8A and viral RNA, which is reduced when PYM1 or MAGOH are suppressed [183]. This suggests that RBM8A, associated to other EJC factors, marks WNV RNA as a NMD substrate, and that Flaviviruses capsid protein interacts with PYM1 to impede EJC-RNA-binding and subsequent NMD.

**Table 5:** NMD evasion trans-strategies of animal viruses.

NMD Inhibition		Virus Classification					
Viral Protein	Mechanism	Realm	Genome ^a^	Family	Genus	Name ^b^	Ref.
N (nucleocapsid)	Interacts with UPF1	Riboviria	+ssRNA	Coronaviridae	Gammacoronavirus	IBV	[179]
		Riboviria	+ssRNA	Coronaviridae	Betacoronavirus	MHV	[157]
		Riboviria	+ssRNA	Coronaviridae	Betacoronavirus	SARS-CoV-2	[180]
C (capsid)	Interacts with UPF1 Interacts with PYM, preventing its interaction with MAGOH and RBM8A	Riboviria	+ssRNA	Togaviridae	Alphavirus	SFV	[181]
		Riboviria	+ssRNA	Flaviviridae	Hepacivirus	HCV	[182]
		Riboviria	+ssRNA	Flaviviridae	Flavivirus	WNV	[183]
		Riboviria	+ssRNA	Flaviviridae	Flavivirus	DENV	[183]
		Riboviria	+ssRNA	Flaviviridae	Flavivirus	ZIKV	[158]
	Interacts with UPF1, promoting its degradation in the nucleus	Riboviria	+ssRNA	Flaviviridae	Flavivirus	ZIKV	[186]
Rex (regulatory)	Unknown mechanism	Riboviria	ssRNA-RT	Retroviridae	Deltaretrovirus	HTLV-1	[187]
Tax (regulatory)	Interacts with INT6 and UPF1, impeding UPF1 association with RNA and promoting phospho-UPF1 sequestration in P-bodies	Riboviria	ssRNA-RT	Retroviridae	Deltaretrovirus	HTLV-1	[188,189]

^a^ Abbreviated genome groups: +ssRNA, positive-sense single-stranded RNA; ssRNA-RT, single-stranded RNA reverse transcribing. ^b^ Abbreviated virus names: IBV, infectious bronchitis virus; MHV, mouse hepatitis virus; SARS-CoV-2, severe acute respiratory syndrome coronavirus-2; SFV, Semliki forest virus; HCV, hepatitis C virus; WNV, West Nile virus; DENV, dengue virus; ZIKV, Zika virus; HTLV-1, human T-cell leukaemia virus type 1.

In addition to its interaction with PYM1, the capsid protein of ZIKV was shown to interact with UPF1 and UPF3B in ZIKV-infected HEK cells [158]. UPF1 depletion in ZIKV-infected NPCs resulted in higher infectious titres and viral RNA levels [158]. Intriguingly, lower protein levels of UPF1, but not of UPF3B, were observed in ZIKV-infected cells [158]. Consistently, in HEK cells expressing ZIKV capsid protein, UPF1 levels were considerably decreased in the nucleus, whereas they were unaltered in the cytoplasm [158]. Nuclear UPF1 levels in cells transfected with the ZIKV capsid protein were rescued by inhibition of the proteasome, indicating that ZIKV capsid protein promotes UPF1 degradation in the nucleus [158]. Expression of ZIKV capsid protein in hepatoma cells showed an accumulation of polyadenylated RNAs in the nucleus, which could also be reproduced by depleting UPF1 from the cells [186]. Taken together, these experiments suggest that UPF1 is involved in mRNA transport to the cytoplasm, and that ZIKV capsid protein disturbs this process by promoting degradation of nuclear UPF1 [186]. Notably, ZIKV infection of NPCs particularly impacts the expression of *FREM2* (FRAS1-related extracellular matrix protein 2), which is a critical neurodevelopmental gene [186]. Knockdown of FREM2 in NPCs resulted in reduced expression of pluripotency marker Sox2 and increased expression of neuronal lineage marker βIII-Tubulin, mimicking human ZIKV infection, and potentially contributing to Zika syndrome [186,190].

Regarding retroviruses, human T-cell leukaemia virus type 1 (HTLV-1) was shown to globally inhibit NMD [187,188,189]. Two different viral regulatory proteins were identified: Rex and Tax [187,188,189]. Rex is an RNA-binding protein, responsible for the export of HTLV-1 unspliced and singly spliced mRNAs to the cytoplasm [191]. Rex can suppress NMD independently from its mRNA export function [187], but the mechanism of NMD-inhibition by Rex remains enigmatic. In contrast, the viral transcription transactivator Tax was shown to directly interact with INT6 (integrator complex subunit 6, also known as eIF3 subunit e) and with UPF1 [188,189]. Co-immunoprecipitation experiments in HEK cells indicated that Tax binding prevents the interaction between UPF1 and INT6, an interaction which is important for NMD [188,192]. Tax was shown to bind to UPF1’s helicase domain and impedes its association with RNA, its ATP hydrolysis, unwinding and translocation activities [189,192]. In addition, immunofluorescence experiments indicate that, in HeLa cells expressing Tax, hyper-phosphorylated UPF1 is sequestered in P-bodies [188]. It was therefore suggested that Tax may inhibit NMD by preventing UPF1’s dephosphorylation and recycling [33,44,188]. Even though Tax interaction with INT6 was shown to be required for Tax-mediated accumulation of UPF1 in P-bodies, the role of INT6 in this process remains unknown [188].

## 5. Concluding Remarks

In recent years, a picture of NMD pathways emerged involving a set of core NMD factors and an increasing number of additional proteins that finetune NMD for specific mRNA transcripts and cellular contexts. These include UPF1 isoforms, UPF3 paralogs, different EJC compositions and specialised NMD factors, such as NBAS and DHX34 during the cellular stress response. This additional complexity, compared to yeast NMD, shows that gene regulation by NMD is a major function of NMD factors in mammalian cells. This function was acquired during evolution in addition to mRNA quality control, the main NMD function in yeast. Mutations in NMD factors (in particular UPF3B) and NMD dysregulation can be associated with neurodevelopmental disease and cancer [193,194]. While NMD functions as an anti-viral defence mechanism against RNA viruses, these viruses have developed diverse strategies to evade NMD, often by directly targeting NMD factors [152,154]. An improved mechanistic understating of NMD will therefore inform the development of novel therapeutic strategies. For instance, tissue-directed gene knockdown by RNA aptamers to inhibit NMD locally has been successfully applied in tumour immunotherapy [143,144]. However, NMD stimulation may be required for other cancer therapies [195].

Interestingly, NMD core factor knockout is embryonically lethal in mice (except for UPF3B), indicating that NMD factors have a role during cell differentiation in early mammalian development [196]. However, research involving knockout or knockdown of NMD factors is complicated by NMD-independent roles of these factors in the nucleus, which affect genome stability, mRNA splicing and export [143,197]. Specifically, UPF1, UPF2, SMG1, SMG5-7 and SMG6 are reported to have functions in DNA damage response and maintenance of telomere integrity, processes which are vital for cells [196]. In particular, UPF1 has numerous roles independent of NMD, e.g., UPF1 regulates cell cycle S-phase progression [197] and replication-dependent decay of histone mRNAs [198], promotes R-loop formation in dsDNA break repair [199], has E3 ubiquitin ligase activity [104], and is involved in other RNA decay pathways, including Staufen-mediated mRNA decay [16]. Taken together, the additional roles and secondary effects of knockdown all complicate the study of molecular mechanisms of NMD factors in cells, requiring innovative approaches to investigate the specific NMD-related functions.

## Figures and Tables

**Table 1:** Missense mutations in UPF proteins found in patients with neurodevelopmental disorders.

Protein	Domain	Gene Variation	Isoform 1	Isoform 2	Related Disease	ClinVar Accession [90]	Ref.
**UPF1**	Helicase	c.2381C > T	T805M	T794M	Autism, developmental delay, joint hypermobility, hypotonia	VCV000996716.1	-
		c.2489A > G	Q841R	Q830R	Intellectual disability	VCV000930204.1	-
**UPF2**	N-terminus	c.91G > T	V31L		Autism spectrum disorder	VCV000996716.1	[132]
**UPF3B**	N-terminus	c.52A > C	T18P	T18P	Syndromic X-linked intellectual disability 14	VCV000287042.5	-
		c.53C > T	T18I	T18I	Syndromic X-linked intellectual disability 14	VCV000698418.4	-
	RRM-L	c.277A > G	M93V	M93V	Syndromic X-linked intellectual disability 14	VCV001012886.1	-
	NOPS-L	c.470A > G	D157G	D157G	Syndromic X-linked intellectual disability 14	VCV001485374.3	-
		c.478T > G	Y160D	Y160D	Syndromic X-linked intellectual disability 14	VCV000011401.3	[125]
	Interdomain	c.520A > G	M174V	M174V	Syndromic X-linked intellectual disability 14	VCV001718760.1	-
	CCL-1	c.667A > G	I223V	I223V	Syndromic X-linked intellectual disability 14	VCV000985848.4	[133]
		c.672A > C	E224D	E224D	Intellectual disability	VCV000981400.1	-
		c.758T > C	I253T	I253T	Cataract, Microcephaly, Severe global developmental delay	VCV000284394.21	-
		c.763A > G	R255G	R255G	Syndromic X-linked intellectual disability 14	VCV001029871.1	-
		c.764G > A	R255K	R255K	Syndromic X-linked intellectual disability 14	VCV000536851.9	-
	Interdomain	c.962T > C	L321S	L308S	Syndromic X-linked intellectual disability 14	VCV000212548.9	-
	CCL-2	c.1049G > A	R350Q	R337Q	Syndromic X-linked intellectual disability 14	VCV001469888.3	-
		c.1072C > T	R358C	R345C	Syndromic X-linked intellectual disability 14	VCV000700934.5	-
		c.1101G > C	K367N	K354N	Syndromic X-linked intellectual disability 14	VCV000804079.4	-
		c.1102C > T	R368W	R355W	Syndromic X-linked intellectual disability 14	VCV001041099.5	-
		c.1103G > A	R368Q	R355Q	Borderline intellectual disability with autism	-	[126]
		c.1117C > T	R373C	R360C	Syndromic X-linked intellectual disability 14	VCV001560636.4	-
		c.1118G > A	R373H	R360H	Syndromic X-linked intellectual disability 14	VCV000547850.2	-
		c.1121G > A	R374H	R361H	Syndromic X-linked intellectual disability 14	VCV000212546.17	-
		c.1136G > A	R379H	R366H	Syndromic X-linked intellectual disability 14	VCV001597732.4	[126]
		c.1201C > T	R401W	R388W	Syndromic X-linked intellectual disability 14	VCV001601042.3	-
		c.1202G > A	R401Q	R388Q	Syndromic X-linked intellectual disability 14	VCV000581972.4	-
